# Supplementary material for: Ethnic differences in healthcare trust and patient satisfaction in England: A cross-sectional survey
Source: PLoS One. 2026 May 27;21(5):e0349884. doi: 10.1371/journal.pone.0349884 (PMC13215503; doi:10.1371/journal.pone.0349884)
Supplement: S1 Table — Survey statements on attitudes and experiences of the NHS. (DOCX) [file pone.0349884.s001.docx]

Supplementary Table S1: Survey statements on attitudes and experiences of the NHS.

| *Item* | *Statement* |
| --- | --- |
|  | ***Communication*** |
| 1 | I believe the NHS is always honest in its communications. |
| 2 | The NHS is frank in its communications to people. |
| 3 | Information on important medical care issues is communicated openly by the NHS. |
|  | ***Competence*** |
| 4 | The NHS is competent in providing a national healthcare service to the people of the UK. |
| 5 | The NHS knows how to supply the healthcare needs of our country. |
| 6 | I feel confident that the NHS can support and provide quality healthcare to the people in the UK. |
|  | ***Ethical care*** |
| 7 | Promises made by the NHS are always delivered. |
| 8 | I can count on the NHS to do things in an ethical manner. |
| 9 | The NHS always put people's interests before its own. |
| 10 | The NHS cares for us. |
| 11 | The NHS has gone out of its way to help people out. |
|  | ***Trust*** |
| 12 | I trust my GP. |
| 13 | I trust the doctors and nurses working in NHS hospitals. |
| 14 | I trust the management that runs the NHS hospitals. |
| 15 | I trust the Department of Health and Social Care that manages the NHS. |
|  | ***Discrimination*** |
| 16 | The quality of care the NHS delivers depends on where you live. |
| 17 | The quality of care the NHS delivers depends on your ethnic background. |
| 18 | The quality of care the NHS delivers depends on your country of origin. |
