# Supplementary material for: Ethnic differences in healthcare trust and patient satisfaction in England: A cross-sectional survey
Source: PLoS One. 2026 May 27;21(5):e0349884. doi: 10.1371/journal.pone.0349884 (PMC13215503; doi:10.1371/journal.pone.0349884)
Supplement: S2 Table — Demographic comparison of ethnic minority respondents with Census 2021 non-White population (England). (DOCX) [file pone.0349884.s002.docx]

Supplementary Table S2: Demographic comparison of ethnic minority respondents with Census 2021 non-White population (England).

| **Characteristic** | **Census 2021 non-White (England)** | **Minority subsample (unweighted)** | **Minority subsample (weighted)** |
| --- | --- | --- | --- |
| Aged 24 years and under* | 41.1% | 34.7% | 33.8% |
| Aged 25 to 34 years | 15.7% | 22.4% | 24.1% |
| Aged 35 to 49 years | 22.9% | 31.6% | 31.7% |
| Aged 50 to 64 years | 13.7% | 6.1% | 4.9% |
| Aged 65 years and over | 6.5% | 5.1% | 5.5% |
| Female | 51.1% | 54.1% | 50.2% |
| Male | 48.9% | 45.9% | 49.8% |

* The census includes children in the “Aged 24 years and under” category, while the survey is based on respondents aged 18 years and over.
