# Supplementary material for: Ethnic differences in healthcare trust and patient satisfaction in England: A cross-sectional survey
Source: PLoS One. 2026 May 27;21(5):e0349884. doi: 10.1371/journal.pone.0349884 (PMC13215503; doi:10.1371/journal.pone.0349884)
Supplement: S3 Table — Full regression results for adjusted models. (DOCX) [file pone.0349884.s003.docx]

Supplementary Table S3. Full regression results for adjusted models.

|  | NHS1 | NHS2 | NHS3 | NHS4 | NHS5 | NHS6 |
| --- | --- | --- | --- | --- | --- | --- |
| Ethnic minority | -0.175 (-0.498, 0.148) | -0.134 (-0.448, 0.179) | -0.447** (-0.753, -0.141) | -0.127 (-0.484, 0.230) | -0.005 (-0.352, 0.343) | -0.025 (-0.388, 0.337) |
| Woman | -0.156 (-0.333, 0.020) | -0.095 (-0.266, 0.077) | -0.191* (-0.359, -0.024) | -0.201* (-0.397, -0.006) | -0.043 (-0.233, 0.147) | -0.202* (-0.400, -0.004) |
| University degree | -0.042 (-0.231, 0.147) | 0.050 (-0.133, 0.234) | 0.007 (-0.172, 0.186) | -0.124 (-0.332, 0.085) | -0.105 (-0.308, 0.098) | -0.234* (-0.446, -0.022) |
| Left–right ideology | -0.103*** (-0.146, -0.060) | -0.111*** (-0.153, -0.069) | -0.092*** (-0.133, -0.051) | -0.139*** (-0.187, -0.092) | -0.170*** (-0.216, -0.124) | -0.091*** (-0.140, -0.043) |
| General trust | 0.156*** (0.100, 0.212) | 0.144*** (0.089, 0.198) | 0.119*** (0.066, 0.172) | 0.172*** (0.110, 0.233) | 0.158*** (0.097, 0.218) | 0.154*** (0.091, 0.217) |
| Age | -0.002 (-0.007, 0.004) | 0.0002 (-0.005, 0.006) | -0.006* (-0.011, -0.0004) | -0.004 (-0.010, 0.002) | 0.004 (-0.002, 0.009) | -0.007* (-0.013, -0.001) |
| Constant | 4.522*** (4.090, 4.955) | 4.402*** (3.983, 4.822) | 5.014*** (4.604, 5.424) | 4.820*** (4.342, 5.298) | 4.704*** (4.239, 5.168) | 4.672*** (4.187, 5.157) |
| Observations | 1,096 | 1,096 | 1,096 | 1,096 | 1,096 | 1,096 |
| R2 | 0.052 | 0.055 | 0.051 | 0.062 | 0.071 | 0.042 |
| Adjusted R2 | 0.047 | 0.05 | 0.046 | 0.057 | 0.066 | 0.037 |
|  |  |  |  |  |  |  |
|  | NHS7 | NHS8 | NHS9 | NHS10 | NHS11 | NHS12 |
| Ethnic minority | 0.042 (-0.262, 0.347) | -0.593*** (-0.891, -0.295) | -0.111 (-0.439, 0.217) | -0.529** (-0.844, -0.214) | -0.174 (-0.492, 0.145) | -0.415* (-0.738, -0.093) |
| Woman | -0.207* (-0.374, -0.041) | -0.076 (-0.239, 0.087) | -0.233* (-0.412, -0.053) | -0.041 (-0.214, 0.131) | -0.120 (-0.294, 0.054) | -0.080 (-0.256, 0.097) |
| University degree | -0.165 (-0.343, 0.013) | 0.097 (-0.077, 0.271) | -0.048 (-0.240, 0.144) | 0.078 (-0.106, 0.262) | 0.032 (-0.154, 0.218) | 0.138 (-0.050, 0.327) |
| Left–right ideology | -0.045* (-0.085, -0.004) | -0.095*** (-0.134, -0.055) | -0.143*** (-0.187, -0.099) | -0.143*** (-0.185, -0.101) | -0.162*** (-0.205, -0.120) | -0.043 (-0.086, 0.0002) |
| General trust | 0.126*** (0.074, 0.179) | 0.166*** (0.114, 0.217) | 0.152*** (0.095, 0.208) | 0.150*** (0.095, 0.204) | 0.142*** (0.087, 0.197) | 0.214*** (0.159, 0.270) |
| Age | -0.004 (-0.009, 0.001) | 0.002 (-0.003, 0.007) | -0.003 (-0.009, 0.002) | -0.002 (-0.007, 0.004) | 0.001 (-0.005, 0.006) | 0.0001 (-0.005, 0.006) |
| Constant | 3.822*** (3.415, 4.230) | 4.569*** (4.171, 4.967) | 4.678*** (4.240, 5.117) | 5.444*** (5.023, 5.866) | 5.168*** (4.741, 5.594) | 4.366*** (3.935, 4.798) |
| Observations | 1,096 | 1,096 | 1,096 | 1,096 | 1,096 | 1,096 |
| R2 | 0.032 | 0.079 | 0.069 | 0.083 | 0.079 | 0.069 |
| Adjusted R2 | 0.026 | 0.074 | 0.064 | 0.078 | 0.074 | 0.064 |
|  |  |  |  |  |  |  |
|  | NHS13 | NHS14 | NHS15 | NHS16 | NHS17 | NHS18 |
| Ethnic minority | -0.429** (-0.740, -0.118) | 0.012 (-0.322, 0.346) | -0.097 (-0.442, 0.247) | 0.147 (-0.181, 0.476) | 0.804*** (0.471, 1.136) | 0.735*** (0.395, 1.076) |
| Woman | -0.054 (-0.224, 0.116) | -0.173 (-0.355, 0.010) | -0.004 (-0.193, 0.184) | 0.171 (-0.009, 0.350) | 0.252** (0.070, 0.434) | 0.267** (0.081, 0.453) |
| University degree | 0.110 (-0.072, 0.291) | -0.108 (-0.303, 0.087) | -0.407*** (-0.608, -0.205) | 0.289** (0.097, 0.481) | 0.150 (-0.044, 0.345) | 0.005 (-0.194, 0.204) |
| Left–right ideology | -0.142*** (-0.184, -0.101) | -0.095*** (-0.139, -0.051) | 0.058* (0.012, 0.104) | -0.048* (-0.092, -0.004) | -0.051* (-0.096, -0.007) | -0.026 (-0.071, 0.019) |
| General trust | 0.164*** (0.110, 0.218) | 0.160*** (0.102, 0.218) | 0.114*** (0.055, 0.174) | 0.007 (-0.050, 0.064) | 0.021 (-0.037, 0.078) | -0.004 (-0.063, 0.055) |
| Age | 0.004 (-0.001, 0.009) | -0.014*** (-0.020, -0.008) | -0.012*** (-0.018, -0.006) | 0.002 (-0.003, 0.008) | -0.015*** (-0.021, -0.010) | -0.013*** (-0.019, -0.008) |
| Constant | 5.171*** (4.755, 5.587) | 4.071*** (3.625, 4.518) | 3.381*** (2.920, 3.842) | 4.761*** (4.321, 5.200) | 3.887*** (3.442, 4.332) | 3.841*** (3.385, 4.296) |
| Observations | 1,096 | 1,096 | 1,096 | 1,096 | 1,096 | 1,096 |
| R2 | 0.09 | 0.068 | 0.037 | 0.019 | 0.088 | 0.061 |
| Adjusted R2 | 0.085 | 0.062 | 0.031 | 0.014 | 0.083 | 0.056 |
|  |  |  |  |  |  |  |
| Note: *p<0.05; **p<0.01; ***p<0.001 | |  |  |  |  |  |
