# Supplementary material for: Ethnic differences in healthcare trust and patient satisfaction in England: A cross-sectional survey
Source: PLoS One. 2026 May 27;21(5):e0349884. doi: 10.1371/journal.pone.0349884 (PMC13215503; doi:10.1371/journal.pone.0349884)
Supplement: Attachment — Submitted filename: response_comments.docx [file pone.0349884.s005.docx]

We would like to thank the two reviewers, María Victoria Martínez López and Peter Goldblatt, as well as the academic editor, Shabnam Iezadi, for the devotion they gave to working through our manuscript. Their detailed comments make clear that they have committed themselves to the improvement of scholarly research and we feel fortunate to be reviewed by such collegial reviewers. As such, we have accepted every suggestion they have made.

Full details are listed below, and the changes can be seen in the “tracked changes” version of the manuscript (though in all honesty, that document will now look a bit of a mess!).

Reviewer comments are in black and our responses are in blue.

**Major revisions**

***Manuscript Title***

1. First, the manuscript's title, "Ethnic Inequalities in Healthcare Trust and Patient Satisfaction in England," implies a nationwide generalizability that is not supported by the cross-sectional survey methodology. Please revise the title to more accurately reflect the study design, for example: "Ethnic Differences in Healthcare Trust and Patient Satisfaction in England: A Cross-Sectional Survey."

This is a good point, and thank you for the suggestion for a new title which we very much agree with. Accordingly we have changed the title of the manuscript to “Ethnic Differences in Healthcare Trust and Patient Satisfaction in England: A Cross-Sectional Survey.”

***Methods***

2. Please provide a clear definition for “minority ethnic groups” used in this study.

We have added extensive discussion of how we define and construct our ethnic minority binary variable in the Methods section. To construct our ethnic minority binary, we follow the standard practice recommended by the UK’s Office for National Statistics (ONS). We also note that the original ethnicity variable provided in our survey is self-reported by the respondents.

3. The minority ethnic share of the sample (10.6%) is substantially below the national figure, which you also mentioned it. Please explicitly state that the analyses are not intended to produce nationally representative estimates. You also may strengthen the robustness section by briefly reporting one or two key weighted vs unweighted comparisons in the appendix or supplementary materials.

We have now made explicit reference to the fact that the analyses are not intended to produce nationally representative samples. We have also pointed out that it is usual in surveys that ethnic minority respondents are disproportionately under-represented and have cited literature to this effect.

We should also point out that the minority ethnic share of the sample is now 8.82% (98 respondents). This was due to respondents who chose “prefer not to say” being incorrectly coded as minority respondents in our earlier code. This has now been corrected and all data in the manuscript have been corrected accordingly. This change did not make any substantive difference to the findings.

On strengthening robustness with weighted and unweighted comparisons: as a robustness check, we ran comparisons across all 18 models with and without weights. These made no difference to our findings at all; indeed, the only differences appeared at the third decimal place. The code to recreate the models with and without weights is now included in the replication file.

4. Treating 7-point Likert scale items as continuous is common practice, but the manuscript does not explicitly justify this choice. Please add a brief methodological justification (with a citation) explaining why linear regression is appropriate for Likert-type data with seven response categories.

We have added a brief methodological justification, with two citations, explaining our treatment of 7-point Likert items as continuous.

5. Although 119 minority respondents is sufficient for basic regression analysis, the study may be underpowered to detect small effects. This limitation is not fully discussed. Explicitly note that non-significant findings should not be interpreted as evidence of no difference, and that limited statistical power may contribute to imprecision.

Just to reiterate our earlier point (under point 3) that we now identify 98 minority respondents. We have added an explicit discussion of statistical power in the Limitations section, noting that while the minority sample size is sufficient to detect medium-to-large effects, smaller differences may not be detectable and null results should not be interpreted as evidence of no difference.

6. Please explain how the participants were recruited, what was the YouGov’s sampling method, and how the informed consents were achieved, in full details.

The Methods section now provides additional detail on YouGov’s panel recruitment, quota-based sampling approach, and opt-in informed consent procedures. Ethical approval details are clarified and confined to the Methods section, in line with journal guidance.

7. Given that ethnicity was assessed via a self-reported online questionnaire without a robust external definition, please discuss how this was handled and the potential implications for the authenticity of results in the limitations section.

We now explicitly note that ethnicity was self-identified and discuss potential implications in the Limitations section, while emphasising that self-identification is standard practice in survey research.

***Results***

8. The Results section would benefit from a clearer introductory overview of the sample and key survey responses and response rate. Please begin the Results section with a brief descriptive summary of the demographic findings, drawing explicitly on Table 1. The descriptive table and its accompanying narrative would be better placed at the start of the Results section rather than in the method section.

We have moved the descriptive table and accompanying narrative to the start of the Results section and added a clear demographic overview drawing explicitly on Table 1.

9. In addition, the authors should consider providing a separate descriptive table summarizing responses to the survey items. This table could report overall mean scores, domain-level scores, and individual item scores, stratified by ethnicity (White vs ethnic minority respondents). Presenting descriptive summaries in this way would help readers contextualize the regression results.

We have added a new descriptive table (Table 2) reporting item-level mean scores and standard deviations for all 18 survey items, stratified by ethnicity (White vs ethnic minority respondents). This table is now presented toward the beginning the Results section to contextualize the regression analyses that follow. The table is organised by conceptual domain (communication, competence, ethical care, trust, and discrimination), allowing readers to assess domain-level patterns without imposing composite scale assumptions. We believe this addition substantially improves transparency and interpretability of the findings.

10. Moreover, you may report descriptive summary scores across key demographic categories (e.g. sex, age groups, education level). These descriptive comparisons would improve transparency and allow readers to better assess patterns in the data prior to the multivariable analyses.

We have further revised the new Table 2 to include descriptive mean scores and standard deviations for all 18 survey items stratified not only by ethnicity, but also by gender (men/women) and educational attainment (university degree vs less than university). Age distributions are reported separately in Table 1 using standard age categories, and age is included as a continuous covariate in all adjusted regression models. We decided against stratifying by age in Table 2 due to space constraints and to preserve table readability.

11. The Results section is generally clear and well organized by domain, which aligns well with the structure of Table 2. However, the narrative would benefit from a more consistent and systematic reporting strategy across domains. For example, for each domain, consider explicitly stating: Whether any ethnic differences were observed overall; Which specific items showed statistically significant differences after adjustment; Whether adjustment materially changed the results. This would help readers quickly grasp patterns rather than focusing on item-by-item details alone.

We have revised the Results section to adopt a more consistent, domain-level reporting structure aligned with Table 2. For each domain (communication, competence, ethical care, trust, and discrimination), we now explicitly summarise whether ethnic differences were observed overall, identify which specific items show statistically significant differences after adjustment, and indicate whether adjustment materially altered the pattern or magnitude of results. This approach allows readers to grasp domain-level patterns before turning to item-level details, while maintaining transparency regarding both adjusted and unadjusted estimates.

12. The narrative places considerable emphasis on unadjusted estimates, with adjusted results mentioned only briefly (“this gap remained significant after adjustment”). Given that adjusted models are central to the authors’ analytic strategy, the results section should give them greater prominence. Please report adjusted coefficients first (or at least alongside unadjusted ones); explicitly note when adjustment attenuates, strengthens, or does not change the magnitude of ethnic differences; avoid implying causal interpretation when discussing adjusted models.

We have additionally revised the Results section to give greater prominence to the adjusted models. For each domain, we now state whether ethnic differences persist after adjustment and explicitly note when adjustment attenuates, leaves unchanged, or does not materially alter the magnitude of observed differences. We have also revised the wording to avoid causal interpretation, framing all adjusted estimates as descriptive associations within this cross-sectional sample rather than as explanatory effects.

13. Confidence intervals are reported approximately in the narrative (“roughly -0.66 to -0.17”), which reduces precision and is inconsistent with the exact values presented in Table 2. Please, report confidence intervals exactly as shown in the table, and use consistent decimal places across coefficients. Additionally, consider including exact adjusted estimates (e.g. “b = -0.39, 95% CI, -0.66 to -0.12”).

We have also revised the Results narrative to report confidence intervals exactly as presented in Table 2 and removed approximate language throughout. All coefficients and confidence intervals are now reported with consistent decimal precision in both the tables and the text. Where results are discussed in the narrative, we now include the exact adjusted estimates and 95% confidence intervals (e.g. b = −0.39, 95% CI −0.66 to −0.12), ensuring consistency and precision across presentation formats.

14. Some interpretive language (like “greater minority scepticism” and “shortfall in transparency”) risks implying broader evaluative conclusions that go beyond the descriptive nature of the results. Please, use more neutral phrasing (e.g. “reported lower agreement” rather than “scepticism”) and reserve evaluative or explanatory language for the Discussion section. Also, emphasize that findings reflect average differences within this sample, not population-wide perceptions.

We have further revised the Results section to remove evaluative or interpretive language and replaced it with neutral, descriptive phrasing. We also now explicitly emphasise that the reported findings reflect average differences within this sample and do not imply population-wide perceptions or causal interpretations.

15. The Results narrative focuses primarily on statistically significant findings, while non-significant results are mentioned only briefly. This may unintentionally suggest selective emphasis. Explicitly state when domains show mostly null findings (e.g. competence).

We have updated the Results section to explicitly report domains in which ethnic differences are largely absent. In particular, we now clearly state when domains (such as competence) show mostly null findings, and note that no statistically significant ethnic differences were observed for the majority of items in those domains in either unadjusted or adjusted models.

16. A few statements in the narrative results do not comply the results of the regression analysis. For example, page 6, line 157, “with both groups generally agreeing at similarly high levels.” Please clarify this.

We have again revised the Results narrative to ensure that all descriptive statements are fully consistent with the regression results and item-level descriptives.

17. A few phrases and statements in the results section are redundant. This information is presented in the methods and available in the tables. For example, “for statements on honesty (“the NHS is always honest in its communications”) or frankness (“the NHS is frank in its communications”)” and “(e.g., “the NHS is competent in providing a national healthcare service” and “I feel confident that the NHS can provide quality healthcare”),”. Please avoid over-repeating the survey items in the results.

We have revised the Results section to reduce redundancy and avoid unnecessary repetition of full survey item wording.

***Table 1***

18. Table 1 provides basic demographic information, but it does not fully describe all key variables used in the regression analyses. Please include descriptive statistics for all demographic and background variables like education, political orientation, etc. Providing these would allow readers to better assess the sample characteristics and contextualize the adjusted models.

We have revised Table 1 to include descriptive statistics for all demographic and background variables used in the regression analyses.

19. Given that ethnicity is the primary independent variable of interest, the descriptive table does not show how other characteristics differ by ethnic group. Please consider presenting descriptives stratified by ethnicity (White vs ethnic minority), either in Table 1 or as a supplementary table. At minimum, indicate whether key covariates (age, gender, education) differ meaningfully by ethnicity. This would help readers assess potential confounding and compositional differences.

We agree that transparency regarding compositional differences by ethnicity is important. We have therefore examined the distribution of key covariates by ethnic group and now explicitly state in the Results section that ethnic minority respondents in the sample are, on average, younger than White respondents, reflecting known demographic patterns in England. We also note that gender and educational distributions differ less markedly across groups. All adjusted regression models include age, gender, and education as covariates, and the reported ethnic differences persist after adjustment. We believe this approach addresses concerns about potential confounding while avoiding unnecessary duplication of descriptive tables in the main text.

20. Age is reported using minimum, maximum, mean, and standard deviation, but this may not fully capture the distribution. Please Consider reporting age categories (e.g. 18–29, 30–44, 45–64, 65+). This would be particularly helpful given the wide age range.

We have added age bands to Table 1.

21. The ethnicity breakdown is clear but minimal. Please add a footnote explaining how ethnicity was measured (self-identified). Reiterate that “ethnic minority” refers to all non-White respondents, and briefly acknowledge heterogeneity.

We have clarified in the main text how ethnicity was measured and operationalised in the study. Specifically, we now explicitly state that ethnicity was self-identified using YouGov’s standard categories, that the “ethnic minority” group refers to all non-White respondents, and that this broad categorisation masks substantial heterogeneity within minority populations. We chose to address these points in the Methods and Results sections to maintain consistency of presentation, rather than duplicating this information in a table footnote.

22. The table is concise but could be improved for readability. Please align labels and values more clearly (e.g. two-column format with “Characteristic” and “Value”). Standardize decimal format (e.g. mean age reported as 50.32 instead of 50·32).

We have revised Table 1 to improve readability by aligning labels and values more clearly using a consistent “Characteristic” and “Value” format. We have also standardised decimal formatting throughout the table (e.g. mean age reported using decimal points rather than middle dots).

23. The total sample size is clearly reported, but it is not repeated in subgroup rows. Consider including N for each subgroup. Ensure that subgroup totals sum exactly to N and explain any discrepancies if present.

We have revised Table 1 to report counts and percentages for each subgroup category, making subgroup sample sizes explicit. Subgroup totals now sum exactly to the full analytic sample (N = 1,111). For one variable (ideology), 13 “prefer not to say” responses are reported as a separate category to ensure totals reconcile fully.

***Table 2***

24. Table 2 contains a large amount of information but is not fully self-explanatory. A reader should be able to understand the table without returning extensively to the Methods section. Explicitly state in the table note that coefficients represent the mean difference in agreement scores (ethnic minority minus White). Specify that outcomes are measured on 7-point Likert scales and clarify the direction of interpretation (higher values = more positive evaluations, except for discrimination items). Indicate that confidence intervals are 95% CIs. You may include this information in the table foot notes.

We have revised the table notes for the regression results (now Table 3) to make the table fully self-explanatory. The notes now explicitly state that coefficients represent mean differences in agreement scores between ethnic minority and White respondents (ethnic minority minus White), that all outcomes are measured on 7-point Likert scales, and that higher values indicate more positive evaluations of the NHS except for the discrimination items (items 16–18), where higher values indicate stronger perceived inequity. The table notes also clarify that all confidence intervals shown are 95% confidence intervals. These revisions allow the table to be interpreted independently of the Methods section.

25. Statistical significance is conveyed using boldface, but this is not explicitly explained in the table notes. Kindly add a footnote explaining that bold values indicate p < 0.05. Consider including asterisks (*, **), which many readers find more intuitive, or ensure consistency with journal style.

We have revised Table 3 to replace boldface with conventional asterisk notation to indicate statistical significance. The table notes now explicitly define the significance thresholds (p < 0.05; p < 0.01; p < 0.001), ensuring clarity and consistency with common journal conventions.

26. Both unadjusted and adjusted results are shown, which is a strength, but the table does not clearly define what variables are included in the adjusted models. Please add a footnote listing all covariates included in the adjusted models (gender, education, political orientation, etc.). Clarify whether all adjusted models include the same covariates.

We have revised the table notes for Table 3 to explicitly list all covariates included in the adjusted models. The notes now state that adjusted models include gender, educational attainment, left–right political orientation, generalized interpersonal trust, and age. We also clarify that the same set of covariates is included consistently across all adjusted models.

27. Items 16–18 differ conceptually from the other outcomes because higher agreement indicates *worse* perceived equity rather than better NHS performance. Please explicitly flag this reversal in the table notes.

We have revised the table notes for Table 3 to explicitly flag this conceptual reversal. The notes now clarify that while higher values indicate more positive evaluations of the NHS for items 1–15, items 16–18 capture perceived inequity, such that higher values indicate stronger perceptions that the quality of NHS care depends on place of residence, ethnic background, or country of origin.

28. Confidence intervals and coefficients are reported with varying precision, which slightly reduces readability. Please standardize decimal places across all coefficients and CIs (e.g. two decimal places). Ensure consistent rounding rules are applied throughout the table.

We have revised Table 3 to standardize the number of decimal places across all reported coefficients and confidence intervals. Consistent rounding rules are now applied throughout the table, improving readability and ensuring uniform presentation of estimates and uncertainty.

29. The table does not report the sample size used for each model. Kindly add a note indicating the total N and the number of minority respondents included in the analyses. Clarify whether all models use the same sample or whether missing data led to varying Ns.

We have revised the table notes for Table 3 to clarify sample sizes for both unadjusted and adjusted models. The notes now indicate that unadjusted models are estimated on the full analytic sample (N = 1,111), while adjusted models are estimated on a slightly reduced sample (N = 1,096) due to missing data on covariates included in the adjusted analyses (left–right political orientation and generalized trust). The number of ethnic minority respondents included in the analyses is also reported. This clarification ensures transparency regarding sample composition and the source of differences in N across models.

30. No information is provided on model fit or explanatory power. Please include R² and adjusted R² for adjusted models in the table footnotes.

We have revised the table notes for Table 3 to report model fit for the adjusted analyses. The notes now report the range of R² (0.019–0.090) and adjusted R² (0.014–0.085) values across adjusted models, providing precise information on explanatory power without overloading the table. Full details are reported in Supplementary Table S1.

**Minor revisions**

31. Ensure consistent use of “NHS” vs “the NHS” in statement wording.

Done.

32. Tables 1 and 2, with accompanying narratives and citations should be placed in the result section.

Also done.

33. Please revise the abstract (particularly result section) according to the change would be made, in the final.

We have revised the abstract to reflect the revised Results section and the additional descriptive and adjusted analyses presented in the manuscript. The Results portion of the abstract now clearly distinguishes between domains in which ethnic differences were observed (ethical care, perceived care, trust in front-line providers, and perceived discrimination) and domains in which no meaningful ethnic differences were found (honesty, promise-keeping, competence, and trust in NHS leadership). The abstract has also been updated to reflect the emphasis on adjusted regression results and to use neutral, descriptive language consistent with the revised Results section.

34. Please also upload study dataset along with your revised paper.

Updated replication data and code posted to the Harvard Dataverse (<https://doi.org/10.7910/DVN/U099DY>) and through the online submission system.

**Additional points for reviewers**

***Additional points for Reviewer 1***

We thank Reviewer 1 for the positive and constructive assessment of the manuscript. In response to the suggestions, we have clarified the conceptual framing of trust by emphasising relational and experiential dimensions of healthcare evaluation, particularly in the Discussion and “What this study adds” section. We have also added trust as a keyword and incorporated relevant conceptual work to situate our findings. While the data do not permit detailed gender-disaggregated or profession-specific analyses, we now explicitly acknowledge these dimensions as important directions for future research and expand the Discussion accordingly. We also elaborate on the policy and institutional implications of the findings, including the relevance of perceived fairness, communication, and discrimination for efforts to strengthen trust in the NHS.

***Additional points for Reviewer 2***

We also thank Reviewer 2 for the careful methodological reading of the manuscript. We have substantially revised the Results section and tables to clearly distinguish between unadjusted and adjusted estimates and to foreground adjusted results throughout. Age is now included as a continuous covariate in all adjusted models, and age distributions are reported using standard categories in Table 1. We have also revised Table 1 to present a more complete and transparent description of the sample, and we explicitly discuss compositional differences by ethnicity (notably age) in the Results. Finally, we clarify attenuation after adjustment and emphasise that adjusted estimates are descriptive associations rather than causal effects.

**Final points**

If there is anything at all that we have missed, please tell us. We will be happy to further revise.
