# Supplementary material for: Ethnic differences in healthcare trust and patient satisfaction in England: A cross-sectional survey
Source: PLoS One. 2026 May 27;21(5):e0349884. doi: 10.1371/journal.pone.0349884 (PMC13215503; doi:10.1371/journal.pone.0349884)
Supplement: Attachment — Submitted filename: response.docx [file pone.0349884.s006.docx]

Dear Dr Iezadi,

On behalf of my co-authors, I would like to thank you and the reviewers for your careful reading of our manuscript and for the constructive and insightful feedback. We have revised the manuscript accordingly and believe that these changes have strengthened both the clarity and robustness of the analysis.

Below we present a detailed, point-by-point response to all comments. All changes have been incorporated into the revised manuscript. Our responses to comments are made in blue.

**Editor**

1. The data analysis method is currently absent from the Methods section, although related information appears in the Results. Please relocate the data analysis description from the Results to the Methods. Specifically, move lines 169–194 on page 10 accordingly.

We have moved the full description of the data analysis procedure from the Results section to the Methods section.

2. Additionally, please transfer the coding strategies to the Methods section and remove them from the Results (lines 153–161, page 7). In the Results, retain only the findings themselves, such as frequencies with percentages and means with standard deviations, as currently presented in the same lines.

We have moved all variable coding descriptions (e.g., construction of binary ethnicity and education variables) to the Methods section. The Results section now contains only descriptive statistics and model-based findings.

3. In the Methods section, please provide a more detailed explanation of how the underlying assumptions of your regression model were tested (for example, linearity, normality, and multicollinearity). Furthermore, elaborate on the specific diagnostic tests used to assess the model's robustness. Clearly indicate whether the results of these tests confirmed that the assumptions were met; if any violations were detected, please describe how they were addressed.

We have added additional information to the Methods section describing how model assumptions were assessed. Specifically:

- Linearity and homoscedasticity were evaluated using residuals-versus-fitted plots.
- Normality of residuals was assessed using Q–Q plots.
- Multicollinearity was assessed using variance inflation factors (VIFs), all of which were low (the highest VIF was 1.18), indicating no concerns.

These diagnostics indicated no substantive violations of model assumptions. Given the ordinal nature of the outcomes, we also note that linear regression is widely accepted and robust in this context.

4. Table 3 revisions:

a. Please also report the total score (mean and SD) for each item in a separate column (first column).

We have added a first column reporting overall mean and standard deviation for each item.

b. Moreover, the statement “Responses are measured on 1–7 Likert scales, where 1 = strongly disagree while 7 = strongly agree” is currently shown in the table’s title. Please move it to the footnote of the table.

The statement describing the Likert scale has been moved from the table title to a table footnote.

c. The “statement Less than Uni” is not a common professional concept please use more professional term like "Less than university degree" or "Below degree level"

This has been replaced with “Less than university degree” throughout.

5. Table 4 revisions:

a. Please use upper limit 95% confidence interval instead of 97.5, and lower limit 95% confidence instead of 2.5.

We now use upper limit 95% confidence interval.

b. Moreover, present the results of unadjusted models in the first column and results of the adjusted models in the second column.

The table has been reordered to present unadjusted models in the first set of columns and adjusted models in the second.

6. In line 228, page 12, please fix the typo “adjust models” and change it to “adjusted models.”

This has been corrected.

7. Please revise the Results section to avoid using words such as "substantially", "significantly", “significantly significant” , or similar terms when the association is not, in fact, statistically significant. Where applicable, use the precise term "statistically significant."

We have systematically reviewed the Results section to ensure that the term “statistically significant” is used only when supported by confidence intervals excluding zero. Ambiguous or overstated language has been revised or removed.

8. In line 259, page 13, please remove the statement: “For the statement ‘The quality of care the NHS delivers depends on where you live’ (statement 16), both White and ethnic minority respondents reported similar levels of agreement.” This is not a relevant result from the regression analysis. You may choose to include it earlier in the Results section where you describe the descriptive findings. However, this statement is not derived from the regression analysis.

The referenced sentence has been removed from the regression-based Results section. The distinction between descriptive and model-based findings has been clarified throughout.

9. In line 227, instead of the statement “with several items showing statistically significant differences,” use a more precise statement like “with two items showing statistically significant differences.”

We have corrected this and have worked through the entire manuscript to make it more specific.

10. In lines 236–238, you have stated: “A similar negative difference was observed for the statement concerning whether the NHS goes out of its way to help people, although the magnitude of the gap was slightly smaller.” The table does not show this relationship was statistically significant. If it is not, please revise the narrative to accurately reflect the result; if it is statistically significant, please edit the table accordingly.

We have revised this passage to ensure consistency with the statistical results. The sentence referring to a non-significant difference has been removed to avoid misinterpretation.

11. Table 1, “Survey statements on attitudes and experiences of the NHS,” is redundant. This information is already provided in Tables 2 and 3. Please move Table 1 to the supplementary files.

This has now been moved to the supplementary files.

**Reviewer 1**

Thank you for carefully considering the reviewers’ suggestions. I believe that the manuscript has now improved significantly as a result of these revisions.

Many thanks to R1 for helping us to make our manuscript better!

**Reviewer 2**

The authors have taken account of the comments from reviewers.

However, in doing so one point has been highlighted. It is that the study is based on 98 responses by those from an ethnic minority - fewer than would be expected in a population sample of this size. The authors need to do more to establish whether the 98 individuals are or are not demographically representative of minoritised ethnic groups. This can easily be done by comparing the characteristics of the 98 individuals to the 2021 non-white Census population using the following link:

https://www.ons.gov.uk/datasets/create

We agree that this is an important issue and have addressed it directly.

We have constructed a new supplementary table (Supplementary Table S2) comparing the demographic characteristics (age, gender, and education) of the ethnic minority subsample with the 2021 Census non-White population in England. This table is now discussed in the Results section. The comparison shows that the subsample is somewhat younger than the Census population, consistent with known demographic patterns, while other characteristics are broadly comparable. We have clarified this in the manuscript and strengthened the discussion of limitations, including the inability to disaggregate by specific ethnic groups due to sample size constraints.
