# Supplementary material for: Ethnic differences in healthcare trust and patient satisfaction in England: A cross-sectional survey
Source: PLoS One. 2026 May 27;21(5):e0349884. doi: 10.1371/journal.pone.0349884 (PMC13215503; doi:10.1371/journal.pone.0349884)
Supplement: Attachment — Submitted filename: response_auresp_3.docx [file pone.0349884.s007.docx]

Dear Dr Iezadi,

On behalf of my co-authors, I would like to thank you again for your help in improving our manuscript. Here are our responses, made in blue.

1) Please add an statement to the method section about the statistical analysis program you used.

We have added this information.

2) Please edit the title of table 3. It still indicates information related to footnote; “Table 3: Means and standard deviations for survey items, stratified by ethnicity. Responses are measured on 1-7 Likert scales, where 1 = strongly disagree while 7 = strongly agree.”, which should be moved to the table’s footnote. You may use an statement like “results of the survey scores on trust in and satisfaction with the NHS in England stratified by minority and non-minority ethnic groups”

We have modified the Table 3 title and added a footnote.

3) Please also revise the title of table 4 and provide more specific and informative title. For example: “results of regression models assessing the relationship between ethnicity and trust in and satisfaction with the NHS in England”

We have changed the title of Table 4.

Again, many thanks for your continued help to improve our manuscript.

Yours sincerely,

Steve Pickering, on behalf of all co-authors.
